# Supplementary material for: AMPK Activation Downregulates TXNIP, Rab5, and Rab7 Within Minutes, Thereby Inhibiting the Endocytosis-Mediated Entry of Human Pathogenic Viruses
Source: Cells. 2025 Feb 24;14(5):334. doi: 10.3390/cells14050334 (PMC11899703; doi:10.3390/cells14050334)
Supplement: Supplementary file 1 [file cells-14-00334-s001.zip › cells-3466875-supplementary.pdf]

Supplementary materials

AMPK activation downregulates TXNIP, Rab5, and Rab7 and inhibits endocytosis-mediated entry of human pathogenic viruses

Viktoria Diesendorf[1#], Veronica La Rocca[1,2,#], Michelle Teutsch[1], Haisam Alattar[1], Helena Obernolte[3], Kornelia Kenst[4], Jens Seibel[5], Philipp Wörsdörfer[4], Katherina Sewald[3], Maria Steinke[6,7], Sibylle Schneider-Schaulies[1], Manfred B. Lutz[1], and Jochen Bodem[1*]

Figure S1: AMPK activation inhibits entry of vesicular stomatitis virus

Figure S2. FACS gating Strategies

Figure S3. Confocal microscopic analyses of the time-dependent downregulation of Rab5, Rab 7 and TXNIP

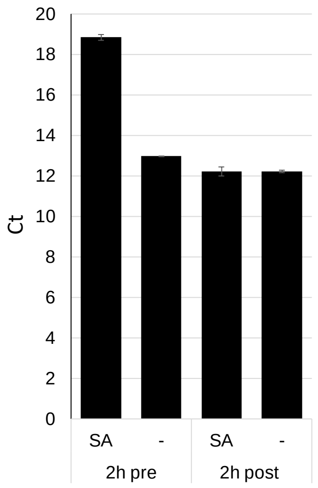

Figure S1: AMPK activation inhibits entry of vesicular stomatitis virus. Vero cells were treated either with 3 mM ASA 2h before or 2 h after infection. Viral RNAs were quantified 16 h after infection with CyberGreen and RTqPCR.

AMPK

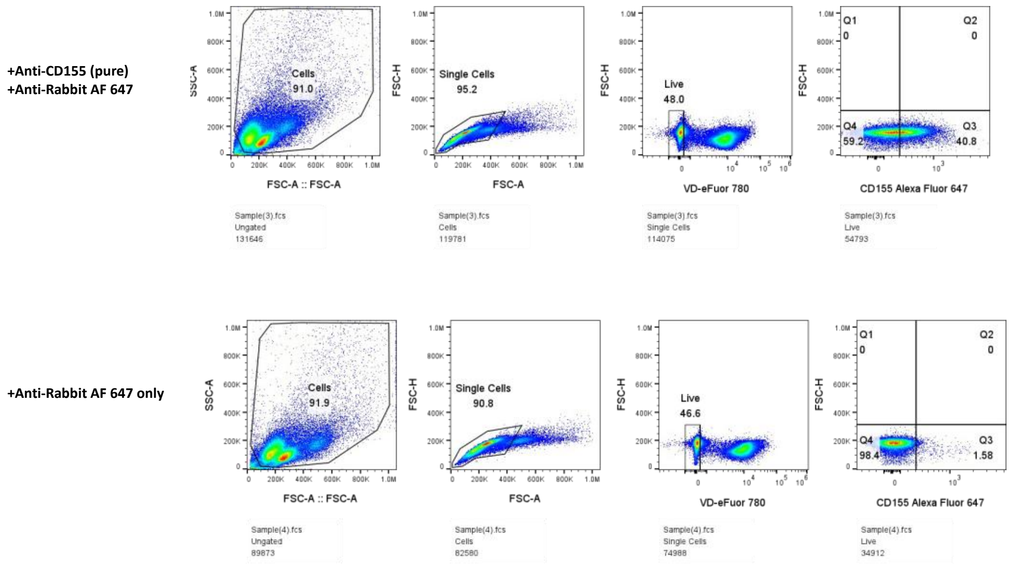

Supplementary materials

ULK

+Anti-CD155 (pure)
+Anti-Rabbit AF 647

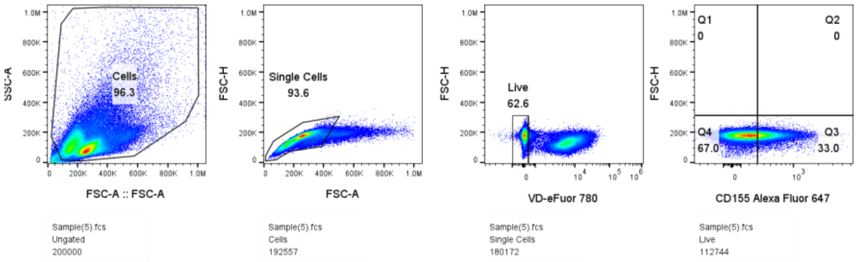

+Anti-Rabbit AF 647 only

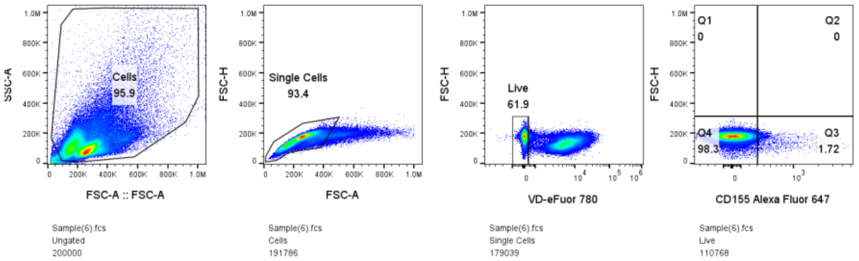

Untreated cells

+Anti-CD155 (pure)
+Anti-Rabbit AF 647

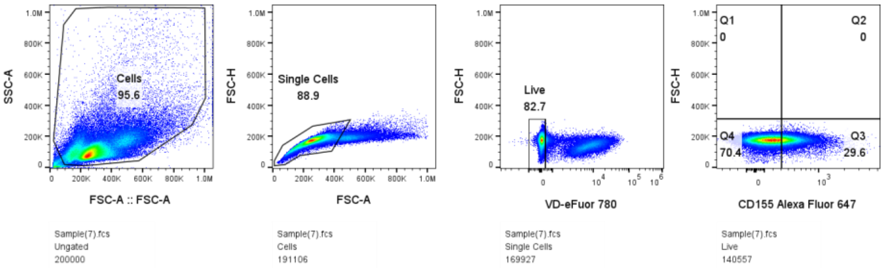

+Anti-Rabbit AF 647 only

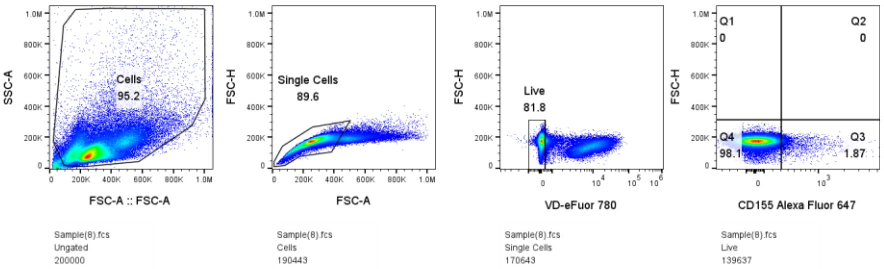

Supplementary materials

Unstained cells

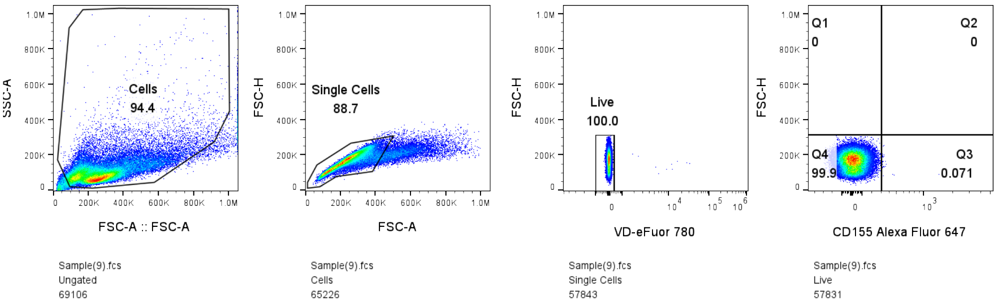

Untreated cells

+Anti-LDL (pure)
+Anti-Mouse PE

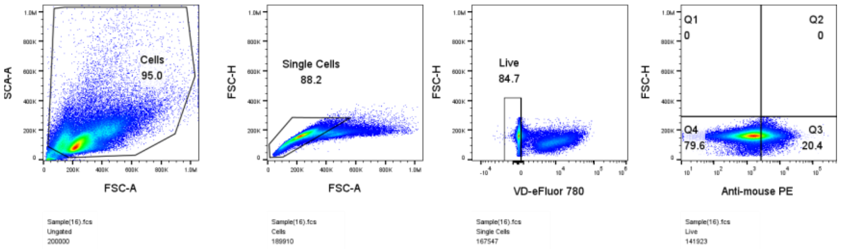

+ Anti-Mouse PE only

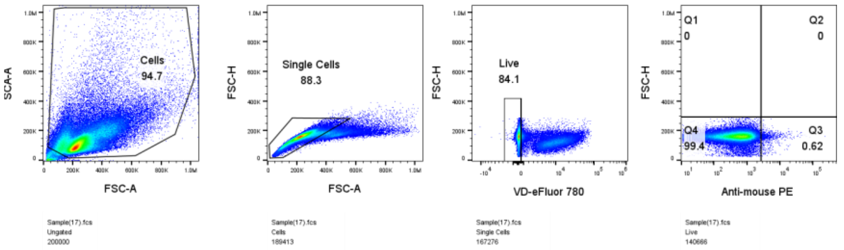

Supplementary materials

Unstained cells

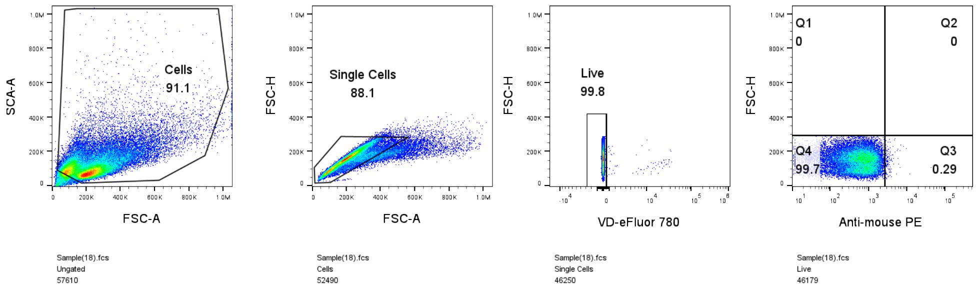

AMPK

+Anti-LDL (pure)
+Anti-Mouse PE

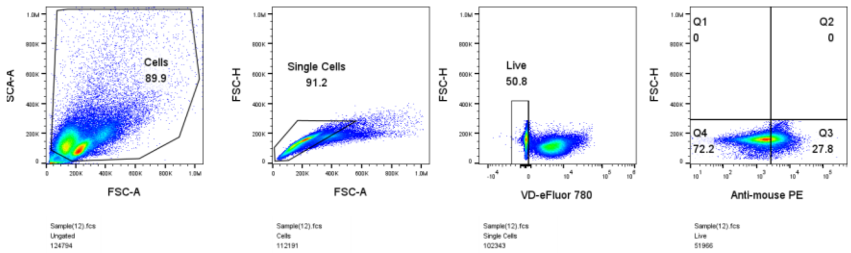

+ Anti-Mouse PE only

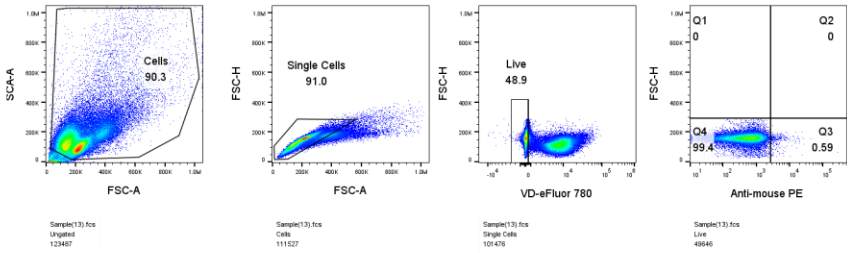

Supplementary materials

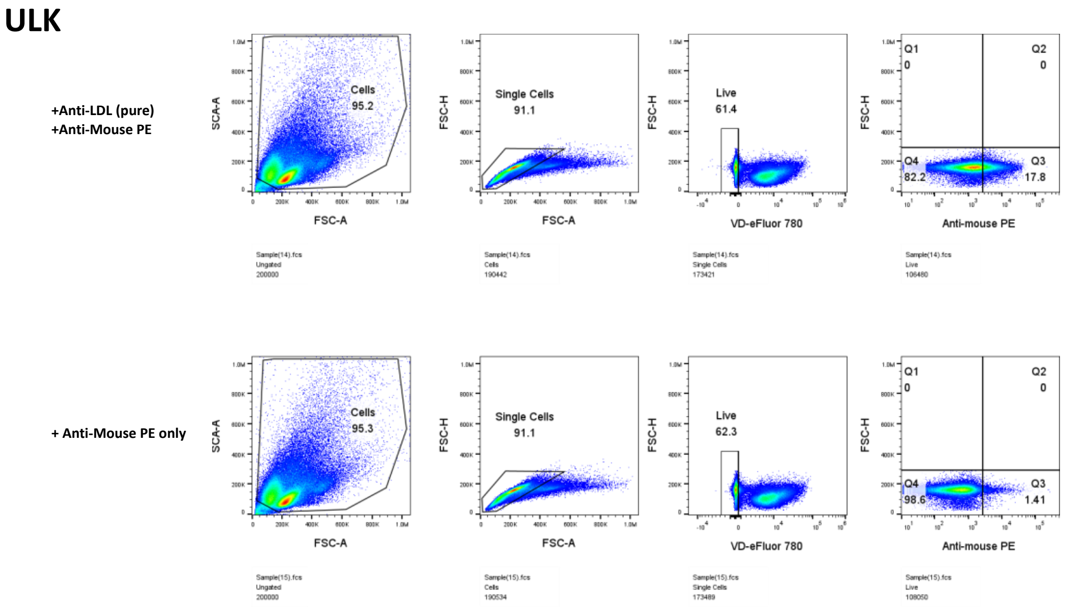

Figure S2. FACS gating Strategies.

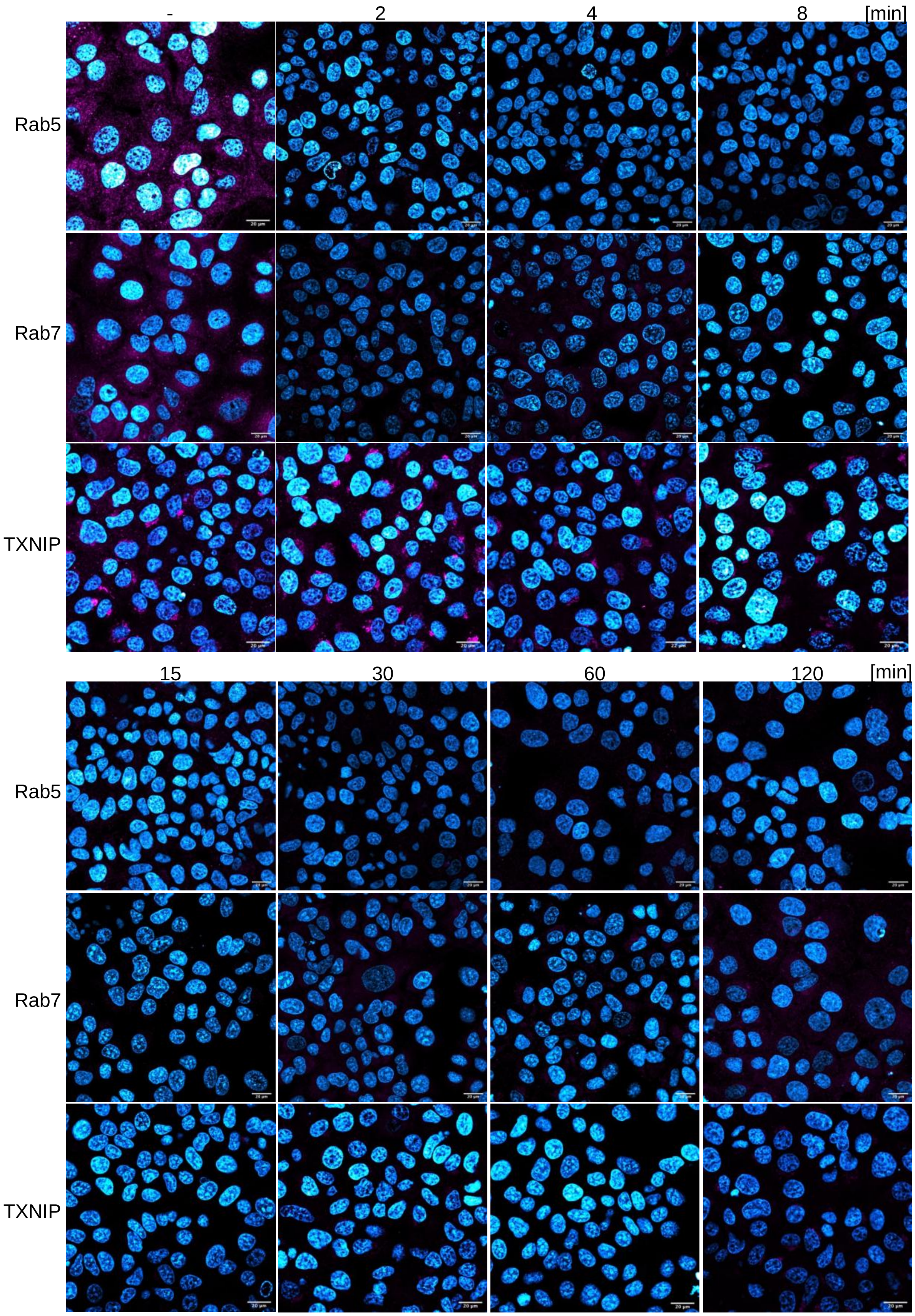

Figure S3. Confocal microscopic analyses of the time-dependent downregulation of Rab5, Rab 7 and TXNIP. Scale bars 20 μm.
